# Digital Health App to Address Disparate HIV Outcomes Among Black Women Living in Metro-Atlanta: Protocol for a Multiphase, Mixed Methods Pilot Feasibility Study

**DOI:** 10.2196/42712

**Published:** 2023-09-15

**Authors:** Rasheeta Chandler, Oluyemi T O Farinu, Dominique Guillaume, Sherilyn Francis, Andrea G Parker, Kewal Shah, Natalie D Hernandez, Synergistic Sisters in Science

**Affiliations:** 1 Nell Hodgson Woodruff School of Nursing Emory University Atlanta, GA United States; 2 Department of Sociology Georgia State University Atlanta, GA United States; 3 Bloomberg School of Public Health Johns Hopkins University Baltimore, MD United States; 4 School of Interactive Computing Georgia Institute of Technology Atlanta, GA United States; 5 Department of Community Health and Preventive Medicine Morehouse School of Medicine Atlanta, GA United States

**Keywords:** Black women, CBPR, community-based participatory research, HIV, human centered design, mhealth, pilot usability study

## Abstract

**Background:**

Cisgender Black women in the southern United States are at heightened risk for HIV and adverse sexual and reproductive health outcomes. Mobile health interventions that target HIV risk while being adapted to the needs and lived experiences of Black women are remarkably limited.

**Objective:**

The study aims to refine *SavvyHER*, a mobile app for HIV prevention, with Black women residing in high HIV incidence areas of Georgia and evaluate the feasibility, acceptability, and usability of *SavvyHER*. This paper describes the procedures implemented to conduct this research.

**Methods:**

Community-based participatory research tenets guide this multiphase study to finalize the development of what we hypothesize will be an effective, sustainable, and culturally relevant HIV prevention and optimal sexual health and reproductive wellness app for Black women. This multiphased, mixed methods study consists of 3 phases. The first phase entails focus groups with Black women to understand their preferences for the functionality and design of a beta prototype version of *SavvyHER*. In the second phase, an app usability pretest (N=10) will be used to refine and optimize the *SavvyHER* app. The final phase will entail a pilot randomized controlled trial (N=60) to evaluate the app’s feasibility and usability in preparation for a larger trial.

**Results:**

Findings from preliminary focus groups revealed educational content, app aesthetics, privacy considerations, and marketing preferred by Black women, thus informing the first functional *SavvyHER* prototype. As we adapt and test the feasibility of *SavvyHER*, we hypothesize that the app will be an effective, sustainable, and culturally relevant HIV prevention, sexual health, and reproductive wellness tool for Black women.

**Conclusions:**

The findings from this research substantiate the importance of developing health interventions curated for and by Black women to address critical HIV disparities. The knowledge gained from this research can reduce HIV disparities among Black women through a targeted intervention that centers on their health needs and priorities.

**International Registered Report Identifier (IRRID):**

DERR1-10.2196/42712

## Introduction

Black women in the United States are disproportionately affected by HIV. In fact, this group accounts for 60% of new HIV infections among women in the nation, despite only making up 15% of the female population [1-6]. A total of 8 out of 10 states with the highest incidence of HIV diagnoses are located within the southern region of the United States (hereinafter called “the South”) [7] and regrettably, Black women also comprise 67% of all HIV diagnoses in this area. Numerous barriers contribute to the increased risk of HIV among Black women which have been well cited in the literature. These barriers often include structural barriers (eg, income inequality, housing insecurity, incarceration, low health care access, and medical racism), social and environmental barriers (eg, small sexual networks, community stigma, intimate partner violence [IPV], and social support), and personal barriers (eg, low knowledge of and access to pre-exposure prophylaxis [PrEP], low self-perceived HIV risk, stigma toward HIV, and medical mistrust). While research often focuses on addressing HIV risk among Black women of lower socioeconomic status, the risk of HIV extends beyond this sub-group. College-educated Black women and Black women of higher socioeconomic status remain at higher risk of HIV due to factors such as limited partner availability, small sexual networks, and health care provider bias. However, they are oftentimes not represented in HIV prevention and larger sexual and reproductive health (SRH) studies [8-10].

Reducing the burden of HIV among Black women requires innovative methods that acknowledge and address the multiple interrelated social and structural factors that promote HIV susceptibility among Black women. PrEP offers an HIV prevention method for women that is discreet, minimizes the need for partner consent, and has no adverse effect on contraception or conception [11-13]. While there is a plethora of PrEP awareness and adoption research that focuses on men who have sex with men (MSM), Black women are overlooked in PrEP literature [14,15]. Studies that have investigated PrEP attitudes exclusively among women have found that while most women have low PrEP knowledge and awareness, they generally respond positively to the idea of using PrEP for HIV prevention [16,17]. Black women’s willingness to consider PrEP depends greatly upon social understandings—whether it is seen as an effective, healthy, and socially acceptable HIV prevention strategy—in addition to cultural and structural factors [18-20]. The heightened risk of HIV acquisition among Black women living in the South combined with the lack of PrEP awareness among Black women confirms the need for innovative, affordable, accessible, and tailored HIV interventions for this group.

Previous research highlights the benefits of using mobile health (mHealth) technology to promote sexual and reproductive health [21-23]. A growing body of literature has reported Black women’s acceptability toward mHealth interventions, including the use of smartphones for health promotion among Black women of reproductive age [19,24-26]. Although Black women are engaged by mobile apps, little to no mobile apps have been developed for the purpose of HIV prevention in Black women [27,28]. Therefore, our research team is developing an mHealth intervention entitled *SavvyHER* (Sexual/HIV Health Electronic Empowerment Resource), a mobile app developed for Black women by Black women which addresses HIV prevention and SRH among Black women living in the South.

## Methods

### Formative Work

**Figure 1:**
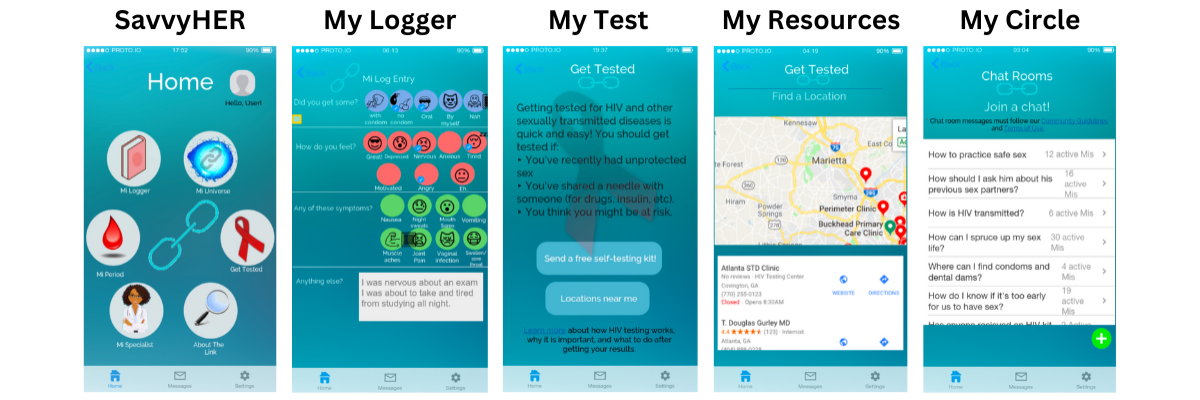
Early depictions of *SavvyHER* features. *SavvyHER*: Sexual/HIV Health Electronic Empowerment Resource.

*SavvyHER* has been developed based on formative work conducted with Black women living in a high-priority region of the South [19]. We solicited input about the most relevant social inequities hindering or prohibiting HIV prevention through focus groups with Black women. This work gathered Black women’s preferences for the functionality, format, and design of a mobile HIV prevention app tailored for Black women. Black women in our formative work specified the following: (1) lack of access to culturally targeted and gender-considerate health care; (2) gender-based power differentials in couple relationships that limit women’s ability to negotiate HIV-protective actions with their male sex partners (eg, condom use); (3) unreliable HIV prevention education and limited access to HIV prevention resources; and (4) susceptibility to other sexually transmitted infections (STIs) and chronic illnesses. Women desired content pertaining to women’s sexual and reproductive health, including HIV prevention, comprehensive health information relative to the health disparities of the Black community, and linkage to women of color health care providers. Preliminary wireframes were developed using findings from our formative work (Figure 1). In addition, the research team conducted a separate study evaluating perspectives from health care providers regarding challenges and facilitators to PrEP initiation among Black women. This work has helped to guide message framing within the preliminary *SavvyHER* app [18].

### Theoretical Frameworks

#### Social Cognitive Theory of Mass Communication

The *SavvyHER* study is grounded in the Social Cognitive Theory of Mass Communication (SCT-MC) [29]. This theoretical framework has been successfully used as a foundation for computer-assisted and internet-based health promotion interventions. SCT-MC applies the SCT to technologically based interventions, postulating that critical mechanisms for behavior change can occur through mass media technology [30]. The constructs of SCT-MC include cognitive factors, situational (ie, environmental) factors, and behavioral factors, with an emphasis placed on symbolic modeling and the notion that people’s perceptions of reality depend upon the media’s symbolic environment, which can contribute to new ways of thinking. Symbolic modeling exerts greater influence in populations that look to media-based images to discern what is and is not reality [31]. SCT-MC also posits that experiences (real or vicarious) facilitate the expansion of knowledge and awareness of causal relationships. Thus, SCT-MC is ideally suited to guide mobile-based interventions focused on behavioral knowledge, behavioral acceptance, and the cultivation of supportive social networks. We will operationalize the SCT-MC to address the health communication priority of using media to improve women’s sexual and reproductive health outcomes, focusing on HIV prevention behaviors*.* As SCT-MC has been used in numerous studies to explore the behavioral acceptance of communications interventions among minority communities, the case can be made for using SCT-MC to evaluate feasibility within a group that has been relatively understudied. Research guided by this framework has identified the benefits of using mobile apps to deliver evidence-based health messaging and training [32,33].

#### Community-Based Participatory Research

This study uses community-based participatory research (CBPR) principles to promote the efficacy, sustainability, and cultural relevance of *SavvyHER* as an HIV prevention app. CBPR is well-suited for addressing health disparities within historically marginalized communities, as it reduces the power dynamic between researchers and participants and fosters shared learning. As a result, CBPR empowers individuals to voice their needs and concerns and facilitates shared learning between researchers and participants [30,34,35]. In order to establish trust and build capacity in our priority community, we ensured that our study team was composed of researchers who identify as Black women to reflect the community we serve. Literature highlights the effectiveness of insider research, or rather, research conducted inside a community to which the researcher also belongs, as a means of “challenging existing structures of power and creating opportunities for the development of innovative and effective solutions to the problems facing society” [36]. In addition to well-established CBPR practices, our team draws on 4 principles of Black feminist epistemology: (1) lived experiences; (2) dialogue; (3) the ethics of caring; and (4) personal accountability [37]. By centering Black women in this project, *SavvyHER* is curated *for and by* Black women to effectively meet this group’s needs [38]. In following CBPR principles, we have assembled a community advisory board (CAB) to steer project priorities, implementation, and dissemination. The multidisciplinary CAB has a total of 8 members and includes HIV clinicians, SRH advocacy representatives, social workers, educators, and nonprofit organizations consisting of Black women.

### SavvyHER App Design and Development

Industrial and technology designers have used human-centered design (HCD) to facilitate innovation [39-41]. Key principles of HCD are the following: (1) the design is driven and refined by user-centered evaluation; (2) the process is iterative; (3) the design addresses the whole user experience; and (4) the design team includes multidisciplinary skills and perspectives. Although not explicitly a research methodology, HCD employs qualitative and quantitative research methods and encourages health innovators to design technology by focusing on how users can, want, and need to use it. This process begins with the identification of health needs, rapid prototyping, and iterative design and development. Once a prototype is created, it is tested and refined based on the user’s experience. The purpose of this approach is to move away from traditional methods of involving users during the latter phases of the prototype and instead regard users as active contributors from the beginning of the design process.

Like other health interventions, mobile health apps face challenges in terms of uptake, engagement, efficiency, and confidentiality. These challenges can be mitigated by ensuring that the intervention content is developmentally appropriate, comprehensible, and comfortable to use. We have chosen an HCD approach to increase the usefulness and use of the app, encourage HIV protective behavior, and promote optimal HIV prevention practices. Additionally, there may be potential risks of abuse during discussions in the app’s virtual peer support communities. The research team will designate chat room moderators to be trained to monitor for abuse. Abuse will be defined in the app-use policies conveyed to participants in the orientation. Examples of these policies might include: no sending of identifying information such as phone numbers or addresses; no posting of links; etc. These policies will be drafted with the CAB. Another potential risk is that women may experience psychological and emotional distress while recounting sexual experiences or discussing sensitive health topics. The research team includes clinicians with experience counseling patients on sensitive health topics. The research team will follow institutional review board (IRB) reporting procedures in the instance that participants experience psychological and emotional distress and will refer participants to local SRH clinicians for support services as needed.

The current version of *SavvyHER* was derived using recommendations from the CAB and based on our preliminary studies [19,42,43]. The app uses text, video, still frames, and other features to convey tailored health messages. The interactive portions of *SavvyHER* are primarily click and swipe functions. Our research team ensured the readability of the app content is no higher than sixth grade level, using the Flesch-Kincaid readability score [44]. *SavvyHER* contains a variety of app features, reflective of the SCT-MC constructs, that were developed based on our formative work with Black women (see Table 1).

**Table 1:** *SavvyHER* mobile app’s features informed by SCT-MC (Social Cognitive Theory of Mass Communication) constructs.

SCT-MC constructs and their descriptions and functions		App feature	Addresses barriers to HIV prevention services
**Personal**			
	Reliable resource for health resources and tools tailored for Black women; addressing Black women’s priority SDOH^a^ issues	*My Resources*	Unreliable or unknown health information sources; HIV risk perception; resource insecurities characterized as SDOH
	HIV prevention plan; health promotion; Health communication messaging	*My Logger*	HIV risk perception: global prevention information that is perceived to be irrelevant to Black women
	Maintain interaction with the user; self-monitoring	*My Logger* *My Resources*	Not the focus of HIV prevention efforts
**Environmental**			
	Digital expertise through conversational interface providing consult regarding reproductive health concerns. Health communication messaging	*My Resources*	Stigma; cultural representation and sensitivity; choice of information dissemination modality, resource accessibility
	GPS locator and geofencing for HIV and women’s health resources; at-home testing for convenience and destigmatizing the sexual health needs of Black women	*My Tests*	Neighborhood health care services; PrEP^b^ providers don’t prescribe to women; stigma
**Behavioral**			
	Messaging, modeling, and learning community	*My Circle*	Social support; subjective norms
	Personalized health messaging based on participant inputs like symptoms, sex behaviors, social stability information (eg, housing and employment status)	*My Logger*	PrEP knowledge and attitudes; HIV testing knowledge and attitudes; and HIV risk perception

^a^SDOH: social determinants of health.

^b^PrEP: pre-exposure prophylaxis.

*My Logger* integrates a self-tracking and preventive health messaging feature that incorporates symbolic modeling, behavioral knowledge, behavioral acceptance, and the cultivation of supportive social networks. Participants are encouraged to journal about any sexual or reproductive health encounters, appointments, indicators of social instability associated with HIV risk behaviors (eg, IPV [45,46] and changes in employment and housing), and descriptive anecdotes in the journal section of *My Logger* [47].

*My Test* consists of two components: (1) a GPS locator for HIV testing and PrEP clinics; we leveraged the existing application programming interface (API) of the HIV.gov testing locator to provide a centralized place for identifying testing and prevention sites; and (2) to make commodity ordering available to participants. Participants can order condoms and STI testing kits through an in-app widget.

*My Resources* gives participants access to a multimedia resource kit consisting of videos, current events, news feeds, podcasts, and support groups related to women’s health, reproductive health, and HIV prevention. In addition, the study team has and will continue to generate health messages, like 1- to 2-minute video clips and infographics.

*My Circle* is a digital environment for women to connect, extending opportunities for peer modeling, learning, and support. In the moderated web-based groups, women share sexual health goals they are pursuing in the app, use messaging features to provide support around these goals and in their lives more broadly, and share insights they have found meaningful through the educational messaging components of the app to support peer learning (eg, through buttons that let them easily share information gained through the *My Logger* and *My Resources* features).

### Study Design

The pilot feasibility trial will consist of 3 distinct phases in which data collection, analysis, and interpretation will occur separately for each phase. The procedures were modeled after other successfully implemented HIV app development procedures [48-50]. The first phase included qualitative focus group discussions (FGDs) with participants; the second phase involved a pilot of the *SavvyHER* app with a select number of participants; and the final phase will consist of a randomized controlled trial in which participants will be randomized to either the *SavvyHER* intervention arm or the control arm. At the time of this manuscript, phases I and II were complete. Phase III will be initiated during second quarter of 2023. This design approach was deemed appropriate as it allows for further refinement of the *SavvyHER* app using the data obtained from phase I and phase II before the randomized controlled trial in phase III.

#### Recruitment and Eligibility

Eligibility criteria are consistent throughout all phases of the study. Participants must meet the following inclusion criteria: (1) be 18-45 years of age; (2) self-identify as Black and African American; (3) be assigned female sex at birth and identify as female; (4) PrEP-qualified based on CDC criteria for residence in high HIV incident areas—reside in Fulton, Cobb, Gwinnett, or Dekalb counties; (5) sexually active within the last 6 months; (6) HIV-negative; and (7) own a smartphone. While the *SavvyHER* app is designed by Black women for Black cis women, we anticipate the unique needs of transgender women. The first iteration of the *SavvyHER* app focuses on cis women; future iterations of the app will incorporate the needs of trans women.

A combination of both passive and active recruitment approaches is used to achieve study enrollment goals for all phases. Passive recruitment involves the distribution of flyers through local SRH organizations, clinics, and social media. Active recruitment efforts incorporate the assistance of SRH clinicians who will refer eligible patients to the study as well as targeted advertisements. All digital and print recruitment communications encompass a QR code—a scannable barcode—that guide interested participants to a web-based questionnaire and a brief description of the study [51]. If eligible, participants provide their contact information (eg, by calling or texting the study phone) for a research team member to contact them to establish an intake meeting for the completion of the baseline assessments and group assignment.

#### Phase I: Focus Group Discussions

Phase I of the study consisted of qualitative data collection through web-based FGDs on Zoom. FGDs were structured in which participants were shown the app and guided through the app’s content and features. After demonstrating the app, participants were asked questions regarding the preferences of each of the *SavvyHER* features, along with questions inquiring about the design of the overall app and features, navigability, and usability.

#### Phase II: App Usability Pretest

Phase II, or the app usability pretest, involved a 1-month pilot test of the *SavvyHER* app with 10 Black women. During a web-based enrollment session, study staff used a checklist to walk participants through procedures for downloading and using the app. Participants were encouraged to use all *SavvyHER* app components over the pilot period, including the HIV and STI test kit ordering feature. Participants completed a weekly questionnaire to provide feedback on the functionality, technical performance, errors encountered, feasibility, acceptability, and overall experience of using *SavvyHER*. Upon completion of the 1-month pretest, participants completed an exit interview through Zoom. Interviews provided further context in regard to the quantitative survey findings to obtain rich insight on app acceptability, feasibility, usability, concerns and challenges in using *SavvyHER*. Exit interviews were audio-recorded and are being transcribed for analysis by the research team. Findings from the app usability pretest will then be used to inform the final version of the *SavvyHER* app for the pilot feasibility trial in phase III.

#### Phase III: *SavvyHER* Pilot Feasibility Trial

After the *SavvyHER* app is refined and optimized using feedback from phase II under the guidance of the CAB, we will evaluate the feasibility, acceptability, and usability of the *SavvyHER* app through a pilot feasibility trial in phase III. This intervention will include a total of 60 Black women who will be recruited and randomized into the intervention group (N=30) or the control group (N=30). The sample size was determined based on feasibility objectives and measures and general guidelines for path analysis, which recommend a sample size of 10-20 participants per study concept while accounting for attrition. Interested participants will provide their contact information, and a research team member will follow up with a baseline assessment and group assignment. After baseline assessments are completed, participants will be randomized 1:1 to either intervention or control using a randomization schedule generated by our statistician using a permuted block procedure [52]. Allocation concealment will be employed before the randomization of participants. Once participants are assigned to groups, blinding will occur, in which researchers involved in data analysis will be blinded to the groups in which participants are enrolled to reduce observer bias and bias in data analysis and interpretation [53]. To restrict access to the *SavvyHER* app to the intervention group, participants will be provided with a single-use registration code that will need to be entered to gain access to the app. Study team members will teach participants how to download the app and navigate app features such as reminders.

Each participant will have 4 months to engage with the app, with e-reminders at time intervals preferred by the participants (a minimum of 3 times per week) through push notifications. We will ask that participants schedule reminders during their peak times of cell phone use. Participants will also be prompted to take a weekly survey to provide app feedback similar to the weekly survey in phase II. Research staff will complete 2-month check-ins through phone call or video chat with participants in both the intervention and control conditions. At month 4, all participants will complete an endline survey in addition to an exit interview. Procedures for exit interviews are the same as those used in phase II. Participants will be remunerated for completion of the baseline assessment and for completion of the endline survey and exit interview. Furthermore, the CAB will review all feedback given and deliberate on what improvements or changes should be made to the *SavvyHER* app.

**Figure 2:**
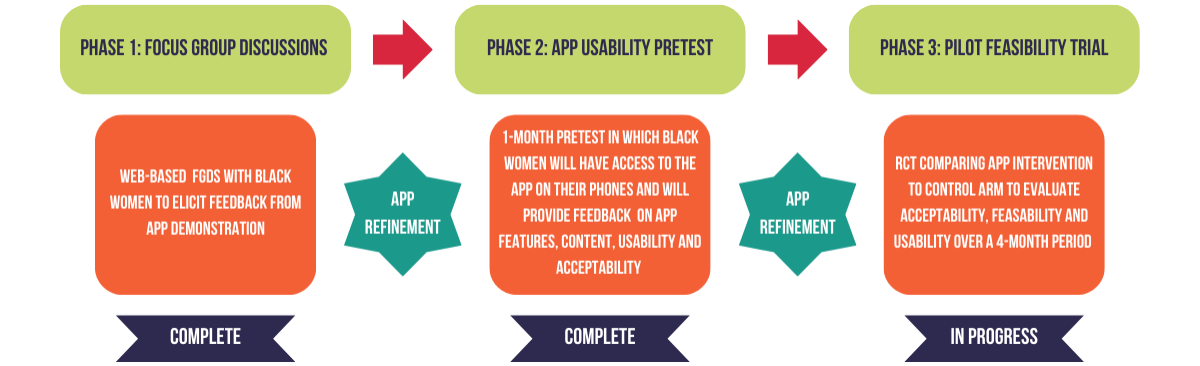
*SavvyHER* study procedures. FGD: focus group discussion; RCT: randomized controlled trial; *SavvyHER*: Sexual/HIV Health Electronic Empowerment Resource.

The control condition will be a 1-time web-based women’s health counseling session with a health care provider. Participants will be given information on (1) STI and HIV prevention; (2) family planning; and (3) general health promotion (eg, exercise and diet). The control condition provides access to HIV prevention materials that are publicly available but that do not offer the dynamic and individually customized features of the health communication and new media research approach proposed herein. The control group will not have access to the *SavvyHER* app until after the study has concluded. See Figure 2 for a visual depiction of *SavvyHER* study procedures.

### Data Collection

All assessments are taken through REDCap, a secure platform for constructing web-based surveys and databases [54]. Participant data is stored under a unique study ID number to ensure security and privacy. In order to inform intervention testing, we collect data on the number and yield of screened participants enrolled, recruiting sources that generated the largest number of enrolled individuals, effective recruitment used for successful enrollment, elapsed time from first contact to enrollment, amount of app interactions, and technological challenges or glitches. Feedback on content and delivery is elicited through REDCap surveys and focus group sessions or interviews, which both undergo qualitative data analysis, also known as coding. Additionally, we collect data on participants’ ownership of smartphones and preferred app formats. These measures will help determine the time, resources, and workload necessary for implementing the HIV prevention mobile app for Black women. Moreover, with this data, we can assess whether users perceived the app as beneficial and whether alternatives (in app content and delivery) can be determined.

All participants provide demographic information through REDCap surveys. Primary outcome measures focus on the feasibility, usability, and acceptability of the *SavvyHER* app in addition to the control group procedures. Feasibility metrics will be determined by meeting predefined benchmarks specific to retention, screening, and interaction with app features. Examples include the number of weekly app sign ins, time spent using the app, and its features. Usability and acceptability will be assessed with the system usability scale (SUS) and a 30- to 45-minute exit interview that queries participants’ experience with study procedures [55]. Because this is a feasibility study, we do not propose a statistical analysis of changes in behavior (eg, PrEP initiation, HIV testing) among users of the *SavvyHER* mobile app. Secondary measures collected in phase III include information on social determinants of health (eg, health care access near her location, employment status, housing status, transportation access, and social support), sexual health history and knowledge, self-perceived discrimination, mental health status, experiences of intimate partner violence, PrEP stigma, racism, and discrimination, personal media and technology use, as well as web-based health information seeking. The completion of the end line survey will also indicate the feasibility of study procedures (eg, retention). A summary of primary and secondary measures is displayed in Table 2.

**Table 2:** *SavvyHER* study outcome measures.

Measure		Questionnaire or survey	Weekly survey	Baseline or end line survey
Demographics		Name, age, race and ethnicity, gender identity, county of residence, relationship status, education, household income	✓	✓
Social determinants of health and women’s sexual health history		Your current life situation [56,57] Protocol for responding to and assessing patients’ assets, risks, and experiences (PRAPARE) [58] A guide to taking a sexual history [59]		✓
**Primary outcomes**				
	Usability/acceptability	System Usability Scale (SUS) [60,61]	✓	✓
	Feasibility	Average number of app sign ins per week; number of minutes spent using the app weekly; sexual health tracking entry, home test kit ordering, number of app glitches; 40% of participants using My Logger, My Test, My Resources and My Circle		✓
**Secondary outcomes**				
	HIV knowledge	Human Immunodeficiency Virus- Knowledge Questionnaire (HIV-KQ AG) [62]		✓
	HIV risk	PrEPa information and self-screening tool for women [63]		✓
	HIV and PrEP stigma, PrEP intention or initiation	Brief HIV PrEP stigma scale (HPSS) [64]		✓
	Self-reported racial/ethnic discrimination	Everyday racial/ethnic discrimination scale [65]		✓
	Depression	Patient health questionnaire-2 (PHQ-2) [66] The patient health questionnaire (PHQ-9) [67]		✓
	Self-reported social media use and technology use; attitudes toward technology use	The media and technology usage and attitudes scale (MTUAS) [68]		✓
	Information seeking	Web-Based Health Information Seeking [69]		✓
	Usability assessment of *SavvyHER*	Health information technology usability evaluation scale (Health-ITUES) [70]		✓
	User experience of *SavvyHER*	User experience questionnaire [71] User experience questionnaire short from (UEQ-S) [71]		✓

^a^PrEP: pre-exposure prophylaxis.

### Data Analysis Plan

#### Quantitative Analysis

Data will be downloaded from REDCap into STATA (StataCorp). Exploratory and descriptive analyses will be conducted for all study variables. Variables will be examined for normality in addition to means, SD, range, and medians. The literature on effect size estimates, from pilot studies and National Institutes of Health (NIH) recommendations, confirms that attempting to obtain valid estimates of effect sizes cannot be statistically justified [72-78]. We have identified feasibility and acceptability as the primary outcomes of this pilot. We have indicated target metrics that will substantiate feasibility, and we will use both quantitative survey and qualitative interview data to determine the acceptability of both the mobile app and the control condition. For all outcome measures (eg, PrEP knowledge), a mean score and 95% CI will be calculated, and distributional assumptions will be assessed. The mean change in outcome measure scores between pretest (T0) and posttest (T1) will be compared (paired samples *t* test) for trends in the expected direction or no change. A paired samples *t* test will be conducted to evaluate the mean change in outcome measure scores between pretest (T0) and posttest (T1) within groups. *t* Tests will also be used to compare variables before the intervention among the 2 groups. To compare differences between intervention and control groups, a 2-way analysis of covariance (ANCOVA) will be used in which covariates will be controlled for. All statistical tests will be performed at a 95% CI with an alpha of .05.

We will use mobile app analytics (eg, Google Analytics) to assess trends in app engagement, such as duration and number of app/module/social media forum engagement, clicks, modules viewed/completed, GPS solicitation, and correct or incorrect responses to questions. Analytics data will be collected as categorical and continuous variables. Continuous variables will be transformed into categorical variables, and multivariable logistic regression will be conducted to evaluate the relationship between usage characteristics and primary outcomes using STATA.

#### Qualitative Analysis

All interviews will be audio-recorded and transcribed using Otter.ai software. Transcribed interview data will be reviewed for overall impressions of the content, materials, activities, and delivery of the *SavvyHER* app. Once transcribed, transcripts will be transferred into Dedoose, a qualitative and mixed methods data analysis platform [79,80]. Our analytic strategy employs a hybrid approach involving concept-driven coding as well as open coding, thus combining the deductive and inductive coding methodologies [81]. Deductive coding denotes the curation of premade codes or themes that are applied to raw data as a means of categorizing quotes for interpretation [82]. Our initial codebook will consist of feedback themes we want to capture regarding the *SavvyHER* app, such as feasibility, acceptability, and usability. Research team members will apply the preset codes to the interview transcripts in an iterative process. Simultaneously, research team members will be creating new codes inductively, or rather, allowing new themes to emerge from the data itself for further analysis [83].

### Ethics Approval

Ethical approval for this study was obtained through Emory University’s IRB (STUDY00002857). Before enrolling in the study, the research team will discuss the purpose of the study, study procedures, and risks and benefits with participants. Participants who agree to participate in the study will provide informed consent to a member of the research team. For the quantitative portion of the study, written informed consent will be obtained from participants. For the qualitative phase, oral informed consent will be obtained. Participants will be provided a unique study ID upon enrollment, and all data will be deidentified to maintain confidentiality. Survey data is also password protected in REDCap. Focus group transcripts and recordings are password protected in digital files. Only study team members on the IRB will have access to data files. Participants will receive compensation for completion of the baseline assessment (US $50) and for completion of the exit interviews (US $80) for a total of US $130.

## Results

Numerous works discuss the gap between scientific discovery and real-world application [84-88]. Literature points to the lack of cultural competency in translational public health research as the source of this gap [88-91]. Our research team and community stakeholders will center on culturally sound, reciprocal knowledge translation, otherwise known as 2-way translation of knowledge. Two-way translation of knowledge involves the creation and distribution of information from academia to the public as well as from the public to academia [91]. In adhering to CBPR approaches, our research team will copresent findings from this project to study participants and other community stakeholders. Disseminating results will not occur for the sole purpose of reporting outcomes but to empower community members to take part in the contextualization of the study findings that guide the development of the *SavvyHER* app. Thus, fostering social action toward mitigating HIV disparities among Black women. The outcomes of the analysis for phase I and phase II will be reported in a future manuscript.

This study was funded in August 2021; data collection is ongoing (we had several phases, and we are currently starting our pilot randomized controlled trial phase now).

The numbers recruited as of submission of the manuscript for this study: a total of 17 participants were recruited for the focus group discussions from December 2021 to March 2022 and met the eligibility criteria to participate. A total of 13 Black cisgender women participated in focus group discussions (phase I). A total of 10 participants enrolled in the study’s single-arm “technology usability pretest.” Among the participants, 8 completed the quantitative and qualitative analyses, which were all components of phase II. We are currently recruiting for phase III, the pilot randomized controlled trial. We aim to recruit 60 (30 in the intervention arm and 30 in the control arm of the study).

The data analysis for the single-arm “technology usability pretest” is currently completed and under review for publication. Furthermore, the data for the pilot randomized controlled trial will be analyzed once the study has concluded. We project that results will be published in the fall of 2024.

## Discussion

### Overview

Although Black women are a priority population concerning HIV in the southern United States, few public health interventions are curated for prevention and treatment among this group. Black women experience some of the highest rates of HIV acquisition due to broader social forces and systems embedded in discrimination. In order to mitigate the disproportionate impact of HIV among Black women, novel and effective strategies must support Black women’s capacity to protect their own health [92]. We anticipate that the *SavvyHER* intervention will address barriers in HIV prevention and broader SRH disparities among Black women by providing access to culturally responsive resources, educational materials, and health services that otherwise would be difficult to acquire given the limited awareness and extensive structural barriers that this community faces. Several studies have begun to explore the benefits of digital platforms in addressing HIV burdens at local and global levels. A review conducted by Cao et al [93] indicated that digital health interventions have demonstrated feasibility in increasing PrEP uptake and optimizing clinical interventions for target communities. It is critical, however, for such interventions to be adequately adapted toward demographic profiles including age, gender identity, sexual orientation, race, and ethnicity [94-96]. As reported by Budhwani et al [97], acceptability testing and adaptation that are underpinned by user feedback significantly increase the likelihood of intervention relevance, satisfaction, and acceptability in new populations of interest. As previously described in our methods, our team will be conducting this study in various phases to ensure that *SavvyHER* is adapted to ensure a strong fit between the intervention and the community being targeted [98,99]. To date, several studies have described the benefits of CBPR in the literature, particularly in creating interventions that reflect community priorities and perceived needs [96-98]. The research teams’ approach of including a diverse array of community stakeholders through CBPR practices, coupled with involving community stakeholders throughout all phases of the research process, can greatly aid in generating awareness and buy-in toward the app on a larger scale [96-98]. Previous literature highlights the need for centering Black voices as a means for discovering knowledge and developing innovative pathways toward advancing health equity [100,101]. The *SavvyHER* mHealth study is spearheaded by Black women for Black women given that they are underrepresented in public health studies as participants and as researchers [102]. By substantively integrating feedback from Black women at the various phases of this study, our team is continuously refining the *SavvyHER* app to ensure that the content, design, and navigation are effective for HIV prevention and treatment among Black women.

### Strengths and Limitations

We have developed a rigorous plan to explore the feasibility, usability, and acceptability of an HIV prevention app for Black women and a strong control comparison. However, potential problems could occur, such as technological glitches, feature imbalances, infirm backend support, irrelevant content, and low engagement, which may be limitations to this study. We have built in questions about all procedures for the focus groups and alternative methods to deal with problems that might arise during the study. This study has low external validity given the randomized controlled trial design and the fact that it is limited to Black cisgender women in the metro-Atlanta area. Therefore, generalization to Black women residing in other areas of Georgia or women of other racial or ethnic identities is limited. This study is strengthened through ensuring that researchers are blinded to which participants are assigned to the intervention and control arms. Thus, observer bias will be mitigated, which will help maximize the validity of the results. Another limitation is the potential for social desirability and recall bias during data collection. Qualitative interviewers will be trained to reduce the likelihood of these biases occurring. While the surveys that will be used contain items from previously validated surveys, the overall survey itself has not been validated, which may contribute to some measurement bias. Measurement bias will be reduced, however, through administering surveys at various time points and coupling Google Analytics data with survey data. Lastly, there may be retention issues with the app; however, we have employed reminders and intrinsic motivation strategies to counter this potential issue.

### Future Directions

Future directions for the *SavvyHER* app involve the integration of mHealth and telemedicine. mHealth solely allows for self-health monitoring, in which app users can take charge of overseeing their health without the guidance of a health care clinician [103,104]. Incorporating telehealth into *SavvyHER* permits not only patient self-monitoring but also clinician-to-patient and clinician-to-clinician interactions. The telehealth structure authorizes the electronic exchange of personal health information that can be used for full-spectrum remote patient care [105,106]. Previous research notes that clinician-level factors affect uptake, as does the adherence support that patients receive [17,107-109]. In forthcoming iterations of *SavvyHER*, we will create clinician-specific functions and coaching that address culturally relevant SRH concerns for Black women. Testing would comprise of a 3-arm study that would assess a control group, a mobile app user-only group, and a group where *SavvyHER* has a health clinician and patient interaction feature.

## Abbreviations

ANCOVA: analysis of covariance
API: app programing interface
CAB: community advisory board
CBPR: community-based participatory research
FGD: focus group discussions
HCD: human-centered design
IPV: intimate partner violence
IRB: institutional review board
mHealth: mobile health
MSM: men who have sex with men
NIH: National Institutes of Health
PrEP: pre-exposure prophylaxis
SavvyHER: Sexual/HIV Health Electronic Empowerment Resource
SCT-MC: Social Cognitive Theory of Mass Communication
SRH: sexual and reproductive health
STI: sexually transmitted infection
SUS: system usability scale

## Data Availability

As this study is currently ongoing, data are not yet publicly available. The authors will make the data available upon request to the corresponding author once the study is completed.
